# Tele-Gastroenterology Midst COVID-19 Pandemic: Patients’ Perspective

**DOI:** 10.7759/cureus.14708

**Published:** 2021-04-27

**Authors:** Zia Rahman, Amjad Ali, Muhammad Usman

**Affiliations:** 1 Gastroenterology, Kettering General Hospital, Kettering, GBR; 2 Internal Medicine, University Hospitals of Leicester, Leicester, GBR

**Keywords:** telemedicine, virtual consultations, gastroenterology, hepatology, covid-19

## Abstract

Objective

The aim of this study was to assess patient satisfaction and experiences of teleconsultation in gastroenterology.

Methodology

Patients who had telephone consultations for gastroenterology and hepatology conditions were contacted, and their responses to questions in a structured questionnaire were recorded. The survey responses were compiled into a Microsoft Excel spreadsheet (2016 version) and formatted using qualitative representation.

Results

A total of 98 patients participated in this survey. The majority of the survey participants were less than 70 years of age (n=69) and more males than females (51 versus 47). Of the patients, 76 (77.5%) were satisfied with their consultations. The positive experiences, as perceived by the participants, were cost, avoidance of travel time and not having to take time off work. The negative experience perceived by the patients was lack of information regarding their condition.

Conclusion

Our study discovered that our patients perceived telemedicine as an effective way of catering for their healthcare needs with good satisfaction rates. This can be used as an adjunct to the traditional face-to-face appointment system to provide uninterrupted healthcare to gastroenterology and hepatology patients during and after the COVID-19 pandemic.

## Introduction

Telemedicine is now well accepted in the wake of the COVID-19 pandemic because it is patient-centred, protects patients and clinicians from viral exposure and provides health outcomes comparable to traditional methods of healthcare delivery without compromising the patient-clinician relationship and enhancing patient satisfaction [1].

The World Health Organization (WHO) defines ‘telemedicine’ as the delivery of healthcare services, where distance is a critical factor, by all healthcare professionals using information and communication technologies for the exchange of valid information for diagnosis, treatment and prevention of disease and injuries, research and evaluation, and continuing education of healthcare providers, all in the interests of advancing the health of individuals and their communities [2]. Telephone clinical services constitute a very important component of telemedicine.

The pandemic has caused serious disruption to the provision of routine healthcare services. According to the British Medical Association (BMA), an estimated 2.47 million to 2.60 million National Health Services (NHS) outpatient appointments were cancelled between April and June 2020 in England alone [3]. To mitigate the disruption in clinical care and limit risk of the infection transmission among the patients and care-givers, major international gastroenterology societies (including the British Society of Gastroenterology and the European Crohn’s and Colitis Organisation) have recommended that elective office visits be conducted remotely, via telemedicine if possible, to decrease density in the office and to provide care to patients who are less willing or unable to travel [4-6].

In line with the advice from the international gastroenterology societies, our hospital, Kettering General Hospital (KGH), started telemedical advice services in gastroenterology outpatient in April 2020. KGH is a district general hospital of the NHS that provides healthcare services to the population of North Northamptonshire and South Leicestershire. The trust carries out an estimated 310,000 outpatients’ appointments each year [7]. Our trust provides tele-audio clinic services currently. Telemedical audio clinic visit involves a clinician calling the patient either on a landline or mobile phone at a predetermined time. We will be using telemedical clinic, teleconsultation and telephone clinics interchangeably while describing this service in this article.

Patients’ satisfaction is important in the acceptance of such teleconsultation services [8] and can be crucial for the successful implementation for long term. Therefore, we designed to conduct a survey to collect feedback data on patients’ satisfaction and experiences with the telemedical services.

## Materials and methods

This patient satisfaction survey covered patients attended gastroenterology and hepatology audio-clinics over two weeks’ period from 8 June till 21 June 2020. All the patients were contacted by clinicians via audio calls on their landlines or mobile phones depending on the preferred way of contact, which they expressed in their basic registration information. Subsequently, calls were made, informed verbal consents (discussed below) were obtained from the patients after explaining to them the study design, methodology and its objective. These audio-calls were made during the working day hours ensuring patients’ ease and comfort.

The study had a cross-sectional design with a convenience sampling technique. We used a 15-item questionnaire (Appendix) to obtain patients’ responses regarding their satisfaction and experiences with audio-telephone consultations. All the questions were read out to them. Options were given to patients either continue with the interview or be called later.

The included questions were mostly binary, i.e. yes, no, unsure and encircle options. Satisfaction scores were measured on a scale from 1 to 5 (<2=poor satisfaction; 2-3=borderline; and 4-5=high satisfaction), and there were options for additional comments. On average, it would take 10 minutes to complete the questionnaire per patient.

All the collected data were compiled into Microsoft (Redmond, WA, USA) Excel spreadsheets (2016 version) that were analysed and results were represented as percentages and graphs using descriptive statistics.

Ethical approval

This study was registered as a quality improvement project and its methodology was deemed compliant to the General Data Protection Regulation (GDPR) by the concerned department in KGH. We were advised by the Research and Development department that no ethical approval was required for this study in which we used anonymised patient data.

Inclusion

Adult patients (>18 years) who had telephone consultation during the aforementioned specified period and who had consented to participate were included in this study.

Exclusion

Patients not consenting to participate, patients not well enough to give feedback and hospitalised patients were excluded from the study.

Consent process 

We initially attempted to obtain a written informed consent using digital signature via Google docs, but not all the study participants had access to the internet. Therefore, informed verbal consent was obtained from the patients who agreed to participate in the study after learning about design, methodology and objectives of the study.

## Results

A total of 197 patients had clinic consultations via telephone audio calls in our Gastro-Hepatology outpatients from 18 June to 21 June 2020; 98 patients consented to participate in this survey, with a respondent turnover of 49.7%. Two-thirds of these patients (n=69; 70.4%) were younger than 70 years (minimum age=26; maximum age=95 years; first quartile=43.25; median=57.50; mean=56.98; and third quartile=70), with more males (n=51) than females (n=47).

The patient demographic characteristics, type of patients (new or follow-up), distance from the clinic and physical disabilities are summarised in Table 1; 78 (79.6%) patients would have needed to drive to the clinic site for a face-to-face appointment, while 17 (n=17; 17.34%) patients would have used alternative transport, and for three (3; 3.06%) patients, the proposed clinic site was within walking distance. Similarly, Table 2 demonstrates that two-thirds of patients had luminal gastroenterology conditions and one-third had liver-related presentations.

**Table 1:** Demographics of the survey participants (patients)

Demographics	Number (%)
Age groups (in years)	
<30	4 (4.08)
31-39	19 (19.38)
40-49	10 (10.20)
50-59	19 (19.38)
60-69	17 (17.34)
70-79	20 (20.40)
>80	9 (9.18)
Gender and clinic exposure	
Males	51 (52.04)
Females	47 (47.95)
New (first clinic audio visit)	32 (32.65)
Follow-up (prior review(s) in face-to-face clinics)	66 (67.34)
Total patients with disability	22 (22.45)
Mobility issues	16 (16.33)
Poor vision	6 (6.12)
Distance from the clinic	
<5 miles	48 (48.98)
5-10 miles	35 (35.71)
10-15 miles	12 (12.24)
>15 miles	3 (3.06)
Transport type	
Driving	78 (79.59)
Lift/taxi/bus	17 (17.34)
Walk	3 (3.06)

**Table 2:** Patients with different health conditions IBD, inflammatory bowel disease

Health conditions	Number (%)
Luminal conditions	64 (65.32)
IBD	27 (27.55)
Irritable bowel syndrome	16 (16.32)
Anaemia	12 (12.24)
Dysphagia	4 (4.08)
Microscopic colitis	2 (2.04)
Chronic diarrhoea	2 (2.04)
Coeliac disease	1 (1.02)
Liver conditions	34 (34.68)
Liver cirrhosis	15 (15.30)
Fatty liver	4 (4.08)
Chronic viral hepatitis	5 (5.10)
Primary biliary cirrhosis	4 (4.08)
Hepatobiliary pain	4 4.08)
Sphincter of Oddi dysfunction	1 (1.02)
Alcoholic hepatitis	1 (1.02)

**Figure 1:**
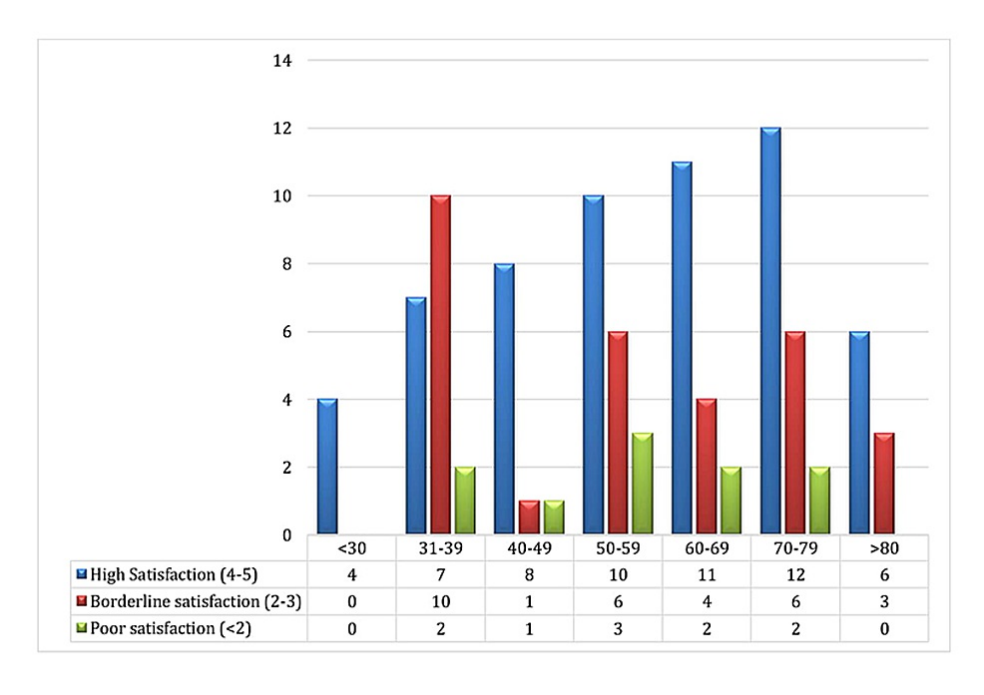
Satisfaction rates in different age groups X-axis represents age groups and Y-axis represents the number of patients in each age group. Colour bars represent different satisfaction rates in each age group as follows: Blue = high satisfaction score (4-5 on Likert scale) Red = borderline (2-3 on Likert scale) Green = poor (<2 on Likert scale)

Three main trends of satisfaction based on Likert scale were noted in our patients, i.e. age groups, with or without prior face-to-face clinic experience (new versus follow-up patients) and presenting symptomatology. As depicted in the bar graph in Figure 1, there is a progressive increase in the satisfaction scores with advancing age of our sampled patients. Only one-third (7/19; 30%) patients in the 31-39 years’ age group reported high satisfaction scores, while three-fifths (12/20; 60%) of patients in the 70-79 years’ age group were highly satisfied with the service.

High satisfaction scores were reported more in patients who had a prior consultation in a face-to-face clinic, i.e. follow-up group (51/66; 77.3%), than patients who had audio-consultation in their first clinic visit, i.e. new patient (15/32; 46.9%).

**Figure 2:**
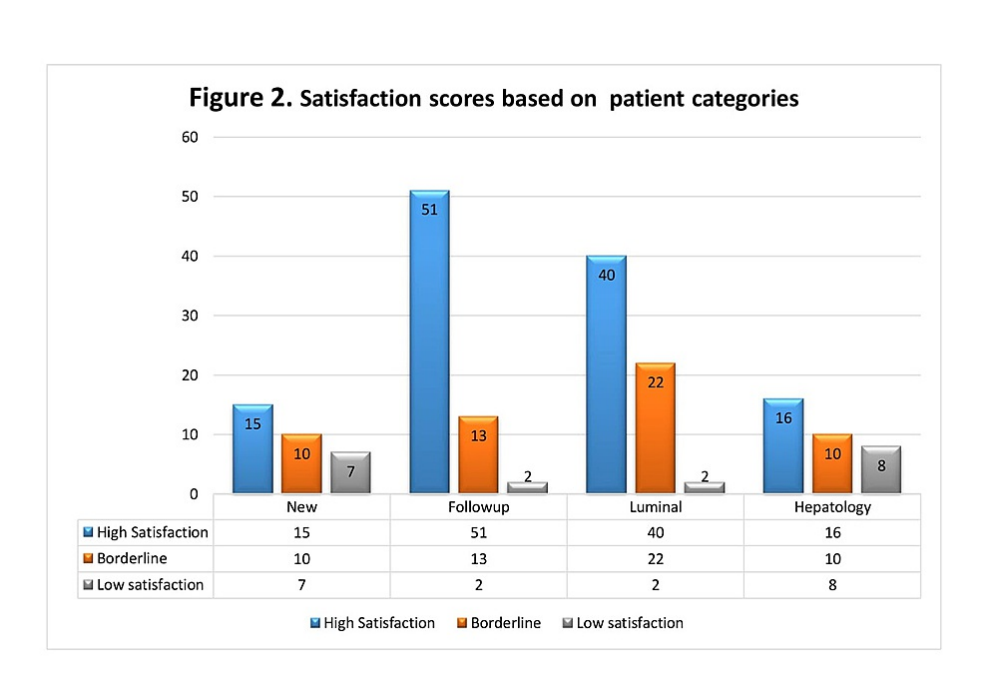
Satisfaction scores based on patient categories X-axis represents patient categories and Y-axis represents the number of patients. Colour bars represent different satisfaction scores as follows: Blue = high satisfaction scores (4-5 on Likert scale) Orange = borderline (2-3 on Likert scale) Grey = poor satisfaction (<2)

Similarly, approval was more in patients with luminal conditions than hepatology conditions. As high satisfaction rates were recorded for luminal gastroenterology patients (40/64; 68.75%) than in liver patients (16/34; 47%). Likewise, low satisfaction scores were seen more among liver patients (8/34; 23.5%) than in luminal patients (2/64; 3.12%). Figure 2 bar charts represent these results.

Over two-thirds (n=68; 69.4%) of the survey patients commented that the telephone consultation saved them time and money, and 47 (47.96%) respondents added that they did not have to take the time off from their workplaces.

A total of 76 (77.5%) patients remarked that their expectations were met, while 16 (16.32%) patients did not think so, and six (6.12%) patients were unsure.

The reported negative experiences were insufficient information and time to ask questions about their conditions, as reported by 25 (26%) patients. This group of patients further commented that they would prefer a face-to-face consultation.

## Discussion

Our survey found that more than two-thirds (77.5%) of the respondents were happy with the use of teleconsultations as a time-efficient and hassle-free method of getting uninterrupted healthcare. Similar trends have been reported elsewhere in the literature. Kruse et al. conducted out a systematic review assessing 44 studies on the use of telecommunication in different hospital and community settings and its effects on factors such as patient satisfaction [8]. The review included studies with varied designs, all of which showed very good satisfaction scores.

An interesting aspect of our study is that 52.6% (10/19) of patients in the 31-39 years' age group were more likely to give borderline scores. Breakdown of this patient group reveals that they were mainly young patients with decompensated liver cirrhosis and hepatitis. Majority of these patients commented that not enough information was given and that the consultation did not meet their expectations. Clearly these patients would benefit more from a face-to-face clinic than a remote consult as physical examination is of paramount importance in such cases. Lower satisfaction scores in younger age group have been reported by Ramaswamy et al. in a respective analysis comparing tele-video and face-to-face clinic consultations in a large academic centre in New York [9].

The telephone consultation was approved relatively more by our elderly (age>70 years) patients, with nearly two-thirds (19/30) giving high satisfaction scores. Feasibility of telemedicine in the older patient population has been reviewed by Batsis et al. They evaluated 17 randomised controlled trials, which implemented telemedicine in patients aged over 65 years across chronic medical conditions. The overall results found that telemedicine was feasible and can be highly acceptable in the older population after addressing the unique barriers including changes to cognition, sensory impairment and access/ability to utilise technology [10].

The acceptability of the service was generally high in luminal and inflammatory bowel disease (IBD) patients in our survey. The majority of these patients expressed that the service has saved them travel time and cost. Similar findings were reported by Li et al., who found that those patients with IBD who completed virtual clinic visits saved time associated with travel and the office visit. Additionally, patients reported less indirect costs associated with the encounter without a decrease in satisfaction with the visit [11].

Our survey results show that new patients (first clinic visit) were less satisfied with the service as compared to those who had clinic reviews (follow up) for their conditions before. Poorer satisfaction scores in the new patients have been reported in a large retrospective analysis [11]. The reason for the finding may be explained by the fact that most of these unhappy patients commented that they were not given enough information and that their expectations from the specialist review were not met.

Our survey shows that more than two-thirds of our study population (69.4%) preferred this method as it saved them travelling cost, travelling time and time off work. These trends have been reported in the literature previously. Bynum et al. found that 92% of patients saved money on travel costs, 84% lost less money due to time lost at work and patients were significantly less likely (p = 0.002) to miss work [12].

The negative experience with our teleconsultation service, as perceived by one-fourth (26%) of our patient cohort, was lack of enough information about their conditions. Nearly one-fifth (17%) of the participants felt that they were unable to ask questions. This is possibly due to the fact that information leaflets could have been provided instantly in a face-to-face clinic, which are not possible with telephone consult. However, this information can be posted either before or after the clinic consultation. These trends in our survey are somewhat different to global trends where the patient barriers are generally around the areas of compliance with the systems and problems with the information technology (IT) system [12,13]. The discrepancy to our survey finding is that we only assessed the audio telephone consultation service rather than video-calling IT system.

Limitations

This was a cross-sectional study based on convenience sampling with a low respondent turnover from a district general hospital; however, clear trends can be seen in the survey findings and can be useful for service structures in other district general hospitals. We obtained informed verbal consents as Google digital signature process would have adversely affected our survey response rates since many of our patients could not access or operate the online consent process. The study also has inherent limitation of lacking the data on the amount of cost and travel time saved, as no specific questions were included in the questionnaire. Another major limitation is that we have undertaken this survey in gastroenterology and hepatology clinics and therefore the results may not be generalisable to other specialties.

Recommendations

Telemedicine has been proven to be very popular among patients with stable health conditions. There is a scope of further expansion of this service in post-pandemic era and can be used alongside face-to-face consultation.

Robust screening with senior clinician triaging system for the allocation of suitable patients to telephone consultation clinics is required as some patients may need to be seen face-to-face ensuring social distancing and taking appropriate infection control measures.

The hospitals need to invest more in upgrading telecommunication services and Information Technology that supports tele-audio and tele-video services such as Zoom, Microsoft teams, Skype, FaceTime video-calling.

We are planning to implement tele-video consultation services in our outpatients, following which we will run another patients' satisfaction survey to evaluate our services.

Provisions should be made to give more information to our patients by posting them leaflets regarding their conditions and encouraging them to access self-help groups. Online information resources can also be directed to that can potentially improve acceptance of the service.

## Conclusions

From the patients’ perspective, teleconsultation is a time-efficient and cost-effective way of providing uninterrupted healthcare in gastroenterology and hepatology clinics during the current COVID-19 pandemic. This method generally has high satisfaction rates among the patients. However, there are patient-centred concerns regarding this method of healthcare provision, which can be addressed using video-consultation to give patients more time to ask questions.

## Appendices

                                          Questionnaire

A)   Demographics

1.       Age:

2.       Gender:

B)    Presentation (please encircle)

3.       New/follow-up (to telephone consultation)

4.       Diagnosis/presenting complaints for clinic review: please specify

C)   Accessibility (please encircle)

5.       Distance from proposed clinic site (miles): <5, 5-10, 10-15, >15

6.       Transport type used: car, bus, lift, walk 

D)   *Patient management (please encircle)*

7.       Did you feel the telephone review met your expectations/needs? Yes/no/unsure

8.       Were you happy with the interaction at the phone clinic? Yes/No

9.       Were follow up plans discussed and were they clear? Yes/ no

E)    Competence (please encircle)

10.   Any physical impairment (mobility impaired, hearing impaired, visual impaired)

11.   Were you happy with interaction with the treating clinician? Yes/no

F)    *Cost and compensation (please encircle)*

12.   Travel time or expense saving

13.   No time off work

14.   Childcare arrangement

G) *  Any additional positive or negative experience*

      Please comment:

H)    *Satisfaction scale:*  Likert scale

             1                               2                              3                    4                           5                                Highly dissatisfied  Dissatisfied           Neutral           Satisfied         Highly satisfied                       
